# Employing an Energy Harvesting Strategy to Enhance the Performance of a Wireless Emergency Network

**DOI:** 10.3390/s22124385

**Published:** 2022-06-09

**Authors:** Ahmed Elshrkasi, Kaharudin Dimyati, Khairol Amali Bin Ahmad, Ezmin Abdullah

**Affiliations:** 1 Department of Electrical Engineering, Faculty of Engineering, Universiti Malaya, Kuala Lumpur 50603, Malaysia; shrkasi@gmail.com (A.E.); ezmin@uitm.edu.my (E.A.); 2 Department of Electrical and Electronic Engineering, Faculty of Engineering, National Defence University of Malaysia, Kem Sg Besi, Kuala Lumpur 57000, Malaysia; khairol@upnm.edu.my; 3 School of Electrical Engineering, College of Engineering, Universiti Teknologi MARA (UiTM), Shah Alam 40450, Malaysia

**Keywords:** disaster management system, clustering technique, D2D communication, energy harvesting

## Abstract

Establishing a wireless communication network (WCN) is critical to saving people’s lives during disasters. Since the user equipment (UE) must transfer their information to the functioning area, their batteries will be significantly drained. Thus, technologies that can compensate for battery power consumption, such as the energy harvesting (EH) strategy, are highly required. This paper proposes a framework that employs EH at the main cluster head (MCH) selected by the enhanced clustering technique (CFT) and simultaneously transmits information and power wirelessly to prolong the lifetime of the energy-constrained network. MCH harvests energy from the radio frequency signal via the relay station (RS) and uses the harvested energy for D2D communications. The suggested framework was evaluated by analyzing the EH outage probability and estimating the energy efficiency performance, which is expected to improve the stability of the network. Compared to the UAV scenario, the simulation findings show that when RS is in its optimal location, it enhances the network EH outage probability performance by 26.3%. Finally, integrating CFT with wireless communications links into cellular networks is an effective technique for maintaining communication services for mission-critical applications.

## 1. Introduction

Diverse unforeseeable disasters such as tsunamis, floods, earthquakes, and torrential rainfall strike many people worldwide. Although disasters are different, disaster management problems are almost identical and repeated, mainly linked to the field complexity, the interoperability problems, and the socio-cultural components. Therefore, communications between functional and dysfunctional areas are significant issues to consider to save people’s lives. Practical communication techniques between first responders and victims rely on the efficient mission-critical transfer of voices and information between victims and first responders [1]. In such circumstances, there are limited resources and services, low reliability and network availability, energy/power loss, and no available communication infrastructures, restricting the implementation of information communications [2].

One of the most commonly shared features of all disasters is the failure of critical communications [3]. The failure of telecommunications infrastructure, whether partial or complete, leads to an inevitable loss of life by causing the delay of disaster relief emergency response. In many scenarios, the breakdown of power networks causes communication networks to disconnect. However, batteries power the communication devices in the disaster region, and their power may eventually run out. Therefore, energy consumption is a significant concern in public safety networks (PSNs). Thus, a PSN needs to implement fewer energy-consuming networks and have the ability to harvest energy.

Energy harvesting (EH) can supply communication devices and wireless networks with the energy harvested. Recently, the EH has become an appealing solution to prolong the wireless networks’ lifetimes. Renewable energy sources such as solar and wind power may provide EH with unlimited environmental energy. Therefore, research in renewable energy has received much attention, notably in cellular communication [4]. Since the robustness and availability of energy are far more critical in a disaster area than in typical situations, using solar panels and wind turbines is not guaranteed to generate the required energy inside the affected area to support the established wireless network. Therefore, ambient radio signals may be potentially viable wireless energy harvesting (WEH) resources, with RF devices converting received signals into energy sources [5,6]. This method is a typical choice for energy-constrained wireless networks. Since emergency energy is mainly restricted, RF energy can be a good solution for power and information transfer.

Researchers have been motivated to examine the simultaneous wireless information and power transfer (SWIPT) and its usefulness in disaster communication methods as a promising technology. SWIPT was developed and allowed energy and data to receivers equipped with RF energy harvesting circuitry. SWIPT can provide continuous and suitable energy needs to wireless networks. In a communications system with SWIPT capability, power transfer and information are simultaneous across wireless media. Consequently, power splitting (PS) and time switching (TS) were proposed as two practical SWIPT strategies in reference [7]. Moreover, the transmission rate optimization problem for a dual-hop multi-relay IoT system with a decode-and-forward (DF) relay supporting the SWIPT technique was investigated by Lu et al. [8].

The essential aspect of our proposed technique is to provide wireless coverage to the disaster area when wireless networks based on the D2D communication technique or unmanned aerial vehicles (UAVs) technique are ineffective due to the considerable distance between the formed clusters or the disaster area size beyond the UAV coverage area limitations. Thus, this paper’s contribution expands the previous work, where the RS-assisted wireless communication network is aided with an energy harvesting strategy to ensure the stability of the network. Besides, it utilized the TS protocol at the main cluster heads (MCHs) that act as a relay node of the cluster to transmit all UE information to BS. Moreover, we enabled reliable connectivity for the RS to MCH and D2D among the clusters communication ranges and calculated the MCH and MCM/SCH power consumptions to ensure that the energy harvested is greater than or equal to at least the power consumption. The system model is expected to perform better using an energy harvesting strategy and become more efficient for disaster-resilient operations.

The rest of this paper is organized as follows: Section 2 summarizes the related work. Section 3 provides a system model for analyzing the energy harvesting technique and D2D performance in the disaster scenario regarding outage probability. Section 4 discusses a disaster recovery framework based on D2D, clustering, and EH. The simulation results and performance of EH and D2D with clustering are detailed in Section 5, while Section 6 concludes the paper.

## 2. Related Work

In critical events such as disasters, the primary intention is to search for and rescue victims. Thus, the importance of communication networks arises to serve in such cases successfully. According to PSN standards, wireless networks are an appropriate alternative for post-disaster relief operations since they are simple to implement in emergencies and do not require pre-existing infrastructure. In the literature, several strategies tailored to various scenarios have been suggested.

In reference [9], the authors proposed using a drone–femtocell technology and constructing an algorithm capable of locating any mobile terminal in a particular monitoring region to search for and identify missing individuals in natural disaster circumstances. This technique uses a series of power measurements based on the reference signal received power (RSRP) to classify the terminal inside or outside the monitoring region. Consequently, even in the presence of obstacles that cause the radio signal’s propagation to be non-isotropic, it roughly determines the position with 1 m accuracy.

In reference [10], the concept of a movable and deployable resource unit (MDRU) was developed by Nippon Telegraph and Telephone (NTT) Corp. The MDRU’s concept is to deploy an entire resource unit to establish a recovery network to the disaster site.

Altay et al. [11] suggested a stand-alone eNode-B architecture that secures service without a backhaul connection by leveraging its own integrated virtual evolving packet core (EPC). The stand-alone eNode-Bs are devised to build backhaul connections, expanding the coverage without a central EPC structure. The stand-alone eNode-B architecture provides improved interoperability and enhances data transmission functionality, particularly in emergencies and disaster events. The work in reference [11] nevertheless did not handle the power consumption problem in the event of a disaster.

Castellanos et al. in reference [12] suggested a capacity-deployment tool for designing and evaluating the backhaul network for UAV-assisted networks in disasters. This tool assigns resources to the ground users and the backhaul network simultaneously, taking into account power limitations and backhaul capacity. They investigated three different backhaul scenarios using 3.5 GHz with carrier aggregation (CA), a 3.5 GHz link, and the 60 GHz band with three different types of drones.

In reference [13], the impact of the relay mobility was handled. The authors evaluated the mobile relay’s capacity and coverage extensions and the impact of mobility on the expected availability duration and route probability establishment. However, the scope of this work is limited to a point-to-point connection with single cells in an assumed idealized circular region. In contrast, we deal with the communication links throughout the multi-hop network in our work, where coverage extends from RS-assisted wireless communication links with clustering techniques to MCHs/SCHs and MCMs/SCMs through D2D links.

In reference [14], a multi-path routing system for PSNs supported by reinforcement learning (RL) and UAV was proposed. The goal is to improve the PSN’s energy efficiency (EE) and thereby increase network lifetime. To begin with, different clustering algorithms are used to generate network configurations. The RL is then used to design a routing topology that considers both the transmission path’s immediate energy cost and its total distance cost.

Since D2D communications allow close UEs to communicate without a base station, this approach may ensure high-speed data transmission and stable, continuous real-time communications. Therefore, In reference [15], the authors suggested a D2D multicast emergency communications technique to make PSN more flexible. This technique is divided into three steps. Firstly, the distance between UEs is used to divide the alternate cluster head. Secondly, there are two types of cluster head selection schemes. One is based on the number of extended UEs, while the other is based on terminal power. Finally, the Hungarian algorithm based on throughput awareness is used for channel multiplexing.

D2D communications and an unmanned aerial vehicles (UAV) approach assisted by D2D links and clustering techniques as an underlay to recover cellular networks in disasters recently attracted much attention. These technologies can help improve energy management, which is a significant concern. Therefore, some recent studies have investigated the possibility of harvesting energy via the RF signals in a cooperative wireless network.

To develop a highly efficient UAV-based wirelessly powered communication network (U-WPCN), the service area for the UAV platform should be selected so that the UAV platform hovers at the appropriate location depending on the positions of the group’s ground terminals, which will be enhanced if the U-WPCN is efficient. Therefore, in reference [16], the authors introduced two networking strategies for maximizing communication performance and improving networking efficiency. The economic strategy uses fewer UAVs while maintaining the required data rate threshold, and the performance strategy uses a higher number of UAVs to enhance the quality of communication (at least one U-IP and one U-EP). They used a greedy algorithm to discover the best hovering point for the area so that the location of the UAV in the network could be determined as an alternative to the conventional local search techniques, such as clustering and genetic algorithms.

In reference [17], researchers proposed a wireless communication system that uses UAV-powered energy harvesting to improve network connectivity and transfer energy during a natural disaster. Furthermore, in reference [18], RF energy harvesting-based power allocation systems were proposed. Researchers investigated a UAV equipped with a pico base station scenario, which might minimize network congestion and traffic overload while enhancing wireless coverage. They used various clustering techniques to overcome energy harvesting challenges contributing to power supply constraints.

In reference [19], a SWIPT approach is suggested to improve energy efficiency (EE) performance and to use radio frequency (RF) signals to harvest energy while functioning with limited battery capacity. A stable matching EH technique is utilized to overcome the problem of resource allocation under the reuse of the spectrum. However, this study does not enhance the EE for D2D communications and cellular networks by improving the CH selection, power transfer, and power splitting ratio.

To address the energy performance constraint, the authors in reference [20] investigated D2D communications based on energy harvesting to maximize the energy efficiency based on the transmit power control and time slot allocation. Thus, a practical resource distribution based on D2D energy harvesting (D2D-EH) was used to improve channel connection quality and reduce the probability of communication outages after disasters.

An integrated strategy for optimal energy harvesting between functional and dysfunctional areas (UAV, CH, and D2D communications) was used. In reference [21,22], UAVs having multiple antennas function as relay nodes to transfer power and transmit information to the UEs located outside the network coverage area. D2D communication within the cluster utilizes an unlicensed spectrum to enhance the system spectrum efficiency for communication between CH and CMs [23]. However, it is challenging to utilize CHs to re-transmit the UAV’s wireless signal to the UEs within its cluster during disasters.

The energy harvesting strategy introduced in this paper could support keeping the wireless network operating during disasters by increasing the UE’s battery life. Thus, the improved clustering technique (CFT) with D2D communication can sustain communication when cellular infrastructure becomes partially or fully dysfunctional. In other words, in a situation where the D2D communications model or the UAV deployment model, which is assisted by the clustering technique to recover cellular networks, could not be utilized due to distance limitations. The proposed framework can be considered optimal when UEs are distributed widely in the disaster area.

## 3. System Model

Mobile wireless communications have unique characteristics that are valuable in disasters. Therefore, wireless links can be easily formed between UEs located in the disaster area within their coverage area or with extended BS-coverage alternatives, as is the scenario introduced in this paper, assuming that the nearest BS to the disaster area is efficiently operational; thus, an ad hoc technique could be used to provide reliable communications via a relay system.

### 3.1. System Description

**Figure 1:**
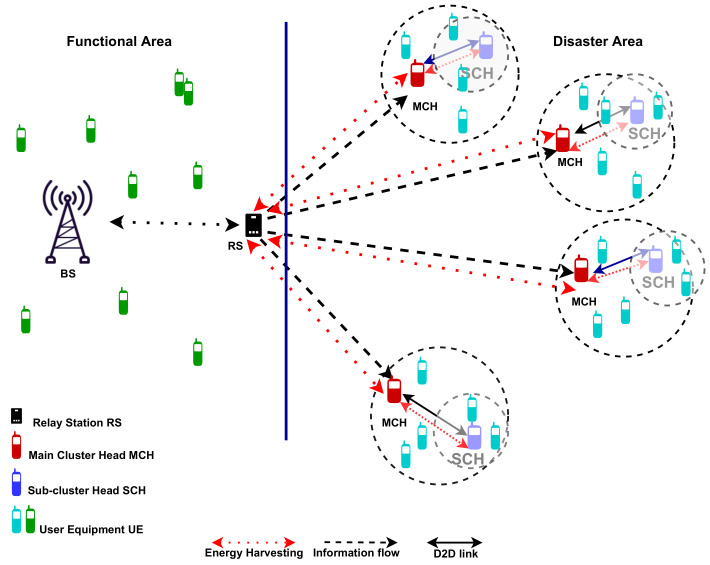
The proposed disasters network framework.

A BS with a fixed energy supply intends to transmit data to UEs located out of coverage (disaster area). We consider a public safety scenario in which impacted people have gathered in secure locations, grouping away from one another, as shown in Figure 1. Due to the distance between clusters being more extensive than the D2D communication range, RS-assisted D2D communication links are impossible. Additionally, UAV-assisted wireless communication links in this context are complex and impractical to execute since the disaster area is larger than the UAV coverage limit. To accomplish the wireless network recovery, we designed a framework in which the RS used wireless communication links to forward BS coverage signals and transfer power to UEs via the cluster head of each cluster.

The main cluster (MC) and sub-cluster (SC) are the two groupings that make up the cluster. The cluster heads for each group were expressed as the main cluster head (MCH) and the sub-cluster head (SCH), respectively. Hence, due to the UE’s selfish nature or a lack of energy supply, the MCH needs to harvest energy before actively assisting since MCH is responsible for relaying the information and transferring power to the UEs that comprise the main-cluster members (MCMs), including the SCH.

Therefore, the following assumptions are taken into account throughout this study.

- A set of N UEs is distributed throughout a radius of a disaster area, based on a homogeneous Poisson point process (PPP) Θ with spatial density $\lambda_{UE}$;
- The mobile relay station RS with spatial density $\lambda_{rs}$ has a fixed energy supply. Furthermore, the decode-and-forward (DF) scheme is the adopted protocol at MCHs to forward the BS information via RS to the UEs;
- The battery levels of UEs are distributed at random in the range of En_min to En_max;
- BS is aware of the last UE’s location and their charge battery level before the disaster occurred and can establish a communication link with the selected UEs (MCHs) in the area via the RS;
- When clusters are formed, each cluster has $I^{k}$ UEs and consists of two groups named MC and SC;
- MCH is an energy-constrained node, and it can use a wireless communications link to harvest energy from RS. Therefore, when energy is harvested from the RS, it is used as a transmitting power to forward information and transfer power to the SCH and other MCMs.

### 3.2. Clustering Formation Technique

The clustering formation technique (CFT) is designed for disaster situations to ensure that all UEs are joined clusters with a predefined cluster radius by calculating the UE distances. Therefore, CM and SC are created within the formed cluster in the first stage of CFT by using Algorithm 1. Whereas the cluster head for MC and SC is selected in the second stage by using Algorithm 2.
**Algorithm 1** CFT stage 1: MC and SC formation**Input:** UEs’ locations and their residual energy at the disaster area**Output:** Formation of main clusters (MC) and sub-clusters (SC)*Initialisation*: Assume that there is a single BS in the operational area. The BS can communicate with all UEs in the disaster area with an RS assist.***Forming MC, and SC.***1:Consider the subset $U_{i}=ni\in UE_{s}$ and $U_{j}=nj\in UE_{s}$2:**for** i=1 to $|\left|U_{i}\right|$3:**for** j=1 to $\left|U_{j}\right|$4:**take**$\;D_{ij}=dist\left(ni,nj\right)$5:**create**$\;MC_{i}=ni\in UE_{s};j\neq i|D_{ij}\leq d1_{D2D}$6:end **for**7:Consider the subset $U_{a}=na\in MC_{i}$ and $U_{b}=nb\in MC_{i}$8:**for** a=1 to $|\left|U_{a}\right|$9:**for** b=1 to $\left|U_{b}\right|$10:**take**$\;D_{ab}=dist\left(na,nb\right)$11:**create**$\;SC_{i}=na\in MC_{i};b\neq a|D_{ab}\leq d2_{D2D}$12:end **for**

**Algorithm 2** CFT stage 2: MCH and SCH selection
**Input:** Residual energy of UEs in MC and its last Location**Output:** Set of selected MCHs1:Initialize MCH = 0..2:

$MCH_{MC-i}=ni\in MC_{i}|E_{MCH}=maxE_{MC_{i}}$

3:
**if** $\;E_{MCH}\geq E_{av}$ $MCH_{MC_{i}}=MCH_{MC_{i}}$


***keep the same UE as MCH***
4:
**if** $\;E_{MCH}<E_{av}$

5:
**create** $\;MCH_{MC_{i}}=ni\in MC_{i}|E_{MCH}=maxE_{MC_{i}}$

6:end **if**7:Sort MCH descendingly based on its residual energy.8:**return** MCH.9:Rerun the previous process whenever the residual energy of the current MCH is less than the threshold value.**Input:** Residual energy of UEs in SC and its last Location**Output:** Set of selected SCHs10:Initialize SCH = 0.11:

$SCH_{SC_{i}}=na\in SC_{i}|E_{SCH}=maxE_{SC_{i}}$

12:
**if** $\;E_{SCH}\geq E_{av}$ $SCH_{SC_{i}}=SCH_{SC_{i}}$


***keep the same UE as SCH***
13:
**if** $\;E_{SCH}<E_{av}$

14:
**create** $\;SCH_{SC_{i}}=na\in SC_{i}|E_{SCH}=maxE_{SC_{i}}$

15:end **if**16:Sort SCH descendingly based on its residual energy.17:**return** SCH.18:Repeat the preceding step when the SCH’s residual energy is less than the threshold value.


#### 3.2.1. CM and SC Forming Stage

The CFT is devised to divide each formed cluster into two groups; MC with a radius of 100 m and SC with a radius of 50 m. However, the number of clusters needed to cover the entire disaster area is determined based on the distance between the UEs available in the disaster area. Therefore, the first stage in forming a cluster is to locate the nearest UE’s distance in a radius of 100 m. Then creates an SC by searching for nearby UEs located inside an MC within a 50 m radius. It is worth mentioning that since the nearest functional BS executes these steps based on the last UEs’ locations, UEs do not consume power. Moreover, data is transferred across a shorter distance between UEs in the SC, reducing the cluster’s power consumption.

#### 3.2.2. MCHs and SCHs Selection Stage

After the cluster formation stage, the MCH and SCH are selected. CFT selection criteria for determining the MCH and SCH are dependent on the residual energy of UEs. Then, the CFT sorts them in ascending order depending on their residual energy. The next sorted UE will act as an MCH/SCH if the residual energy of the current MCH/SCH goes below the defined threshold. The energy threshold value is estimated as $E_{th}=\sum_{n=1}^{N}E_{n}/N$, where *N* is the UEs’ total number per cluster and $E_{n}$ is the UEs’ residual energy. Furthermore, each MCH sends an acknowledgment to MCMs and SCHs within a cluster. Similarly, SCH acknowledges UEs that join a sub-cluster (SCMs). Each of the SCMs and MCMs send a join request to the designated MCH/SCH. To avoid interference, SCH generates a TDMA schedule for SCMs to send data over D2D links, while MCMs use the D2D multi-hop link.

### 3.3. Time Switching-Based Protocol

**Figure 2:**
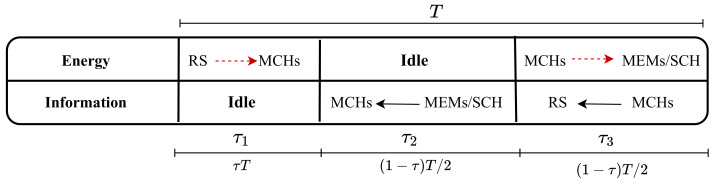
Time switching-based protocol.

In order to transmit information and transfer power to MCMs, including the SCH, the time switching-based protocol (TS) was implemented at each MCH. The total symbol duration (T) is partitioned into three intervals with lengths of $tau_{1}$, $tau_{2}$, and $tau_{3}$, where $tau_{1}+tau_{2}+tau_{3}=1$, as illustrated in Figure 2. T denotes the time required for a specific block of information that can be transmitted from the RS to the MCH. Specifically, each interval has the lengths of τT, (1−τ)T/2, and (1−τ)T/2, respectively, where 0 < τ < 1 denotes the time switching ratio.

Figure 2 shows the energy transfers from RS to the MCH during the first time slot with a duration of $\tau_{1}$ T. The information is transmitted from MCMs/SCH to MCH in the second time slot $\tau_{2}$ T, while in $\tau_{3}$ T, the MCH transfers energy to the MCMs/SCH and transmits information to RS. There is no direct link between the RS and the MCM/SCH. The first interval corresponds to the energy harvesting phase at the MCH, during which the RS wirelessly transfers its energy to the MCH with power $P_{n,RS.1}$. Thus, the total energy harvested at the MCH is estimated for each block by

(1) $$E_{i}=\tau_{1}T\eta \sum_{n=1}^{N}P_{n,RS.1}{\left|H_{n,RS-MCHi}\right|}^{2}$$

where η denotes the energy harvesting efficiency as 0≤η≤1. The information transmitted from the MCMs/SCH to the MCH is represented by the second phase of duration $tau_{2}$. Furthermore, the MCH forwards the signal to the RS and energy to the MCMs/SCH in the third phase of the transmission block $tau_{3}$. Since the bandwidth is divided into *N* orthogonal sub-carriers, n∈1,2,⋯,N, $P_{n,RS.1}$ expresses the transmitting power of the RS across the *n*th sub-carrier for the energy transfer. Moreover, $H_{n,RS-MCH_{i}}$ indicates the channel gain between the RS and MCHs. The RS shall allocate all available power to the sub-carrier with the highest channel gain to optimize the harvested energy at the MCH. Therefore, EH could be expressed as:

(2) $$E_{i}=\tau_{1}G_{i}$$

where

(3) $$E_{i}=\tau_{1}T\eta Pmax_{i}{\left|H_{n,RS-MCHi}\right|}^{2}$$

where $P_{max}$ denotes the RS’s maximum transmit power for information and energy transfer. As a result, $P_{i}⩾\sum_{n=1}^{N}P_{n,RS.1}$. The signals are transmitted to the MCH via the RS through the *N* sub-carrier in the second slot. Once signals are received, the MCH decodes them, distributes them to different sub-carriers, and forwards them to the MCMs, including the SCH. Therefore, the relay network’s highest feasible end-to-end data rate is obtained as in reference [24].

(4) $$\begin{array}{cc} R_{i}= & min(\tau_{2}\sum_{n=1}^{N}log_{2}\left(1+P_{n,RS.2}\times \gamma_{n,RS-MCHi}\right),\\ & \tau_{3}\sum_{n=1}^{N}log_{2}\left(1+P_{n,MCHi}\times \gamma_{n,MCHi-MCMj}\right) \end{array}$$

where $P_{n,RS.2}$ and $P_{n,MCHi}$ indicate the transmission power of the RS and MCH for the transmission of information over the *n*th sub-carrier, respectively.

(5) $$\gamma_{n,RS-MCHi}={\left|H_{n,RS-MCHi}\right|}^{2}/\sigma_{0(MCH_{i})}$$

and

(6) $$\gamma_{n,MCHi-MCMj}={\left|H_{n,MCHi-MCMj}\right|}^{2}/\sigma_{0(MCH_{j})}$$

where $\sigma_{0(MCH_{i})}$ and $\sigma_{0(MCH_{j})}$ are the noise powers over each sub-carrier at the MCH and MCM, respectively. The energy harvested in the first time slot should be not less than the energy consumed to transmit the MCHs’ information [25,26,27], which can be expressed as:

(7) $$E_{i}\geq \tau_{3}T\sum_{n=1}^{N}P_{n,MCHi}$$

It is expected that many UEs would receive RS signal coverage, which could be candidates as MCHs. Therefore, the CFM was introduced to ensure the joining of all UEs in the disaster area to clusters, besides optimizing the power consumption and enhancing the network capacity. The CFM is an essential step toward communicating before information and energy can be transmitted in such a scenario.

## 4. Power Transfer for the Proposed Clustering Network

**Figure 3:**
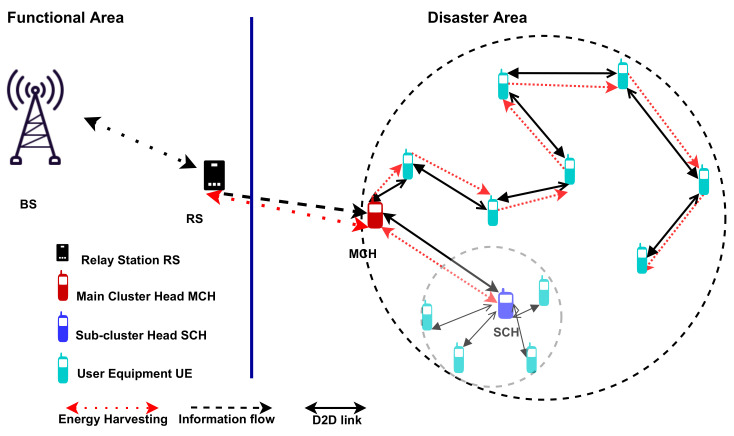
Energy transfer scheme within a cluster.

This section investigates the intensity of the signal carried from the RS to the MCH, then from the MCH to the MCMs/SCH, and the possibilities of harvesting energy in emergency circumstances. D2D communication between the MCH and MCMs/SCH is utilized to increase the range of RS coverage and improve the quality of service (QoS), including energy efficiency. Therefore, the energy harvesting performance is evaluated within each cluster’s D2D communication range while considering the cluster architecture, as shown in Figure 3. We studied a scenario in which the RS transmits an RF signal and its associated information to MCHs through wireless links, and each MCH then communicates with the MCMs/SCH connected to it in the D2D communication range. It is worth noting that the SCMs were excluded from harvesting energy since they were so close to the SCH that only a tiny amount of energy was required to send their information to the SCH. In order to enhance the MCH throughput, the RS transmits the main beam to the MCHs, and then the received energy can be harvested by the MCH and transmitted via D2D communication to the MCMs/SCH. In a disaster, a device’s energy consumption is critical for rescue teams to save lives. We expect that CFT-based energy harvesting could provide a more efficient and stable wireless network.

### 4.1. Performance Analysis of D2D in Clustering

Considering that, there are $C_{k}$ clusters formed, where k=1,2,⋯K, as shown in Figure 1. Within each cluster $C_{k}$ there are two groups of MCs and SCs. In the MCs there are $I_{MC}$ + 1 UEs: one MCH $n_{MC,0}$, $I_{MC}$ MCM UEs $n_{MC,i_{MC}}$, and $i_{MC}$ = 1,2,, $I_{MC}$, while in the SCs there are $I_{SC}$ + 1 UEs: one SCH $n_{SC,0}$, $I_{SC}$ SCM UEs $n_{SC,i_{SC}}$, and $i_{SC}$ = 1,2,, $I_{SC}$. Therefore, the estimate of the time required to transfer data consisting of $S_{T}$ bits from the *i*th $MCH_{i}$ to the *k*th $MCM_{k}/SCH_{i}$ links with an achievable rate of $R_{i,k}$ bps is $ST/R_{i,k}$. Moreover, the $MCM_{k}/SCH_{i}$ battery power is drained by $P_{Rx,i,k}$ for receiving data from $MCH_{i}$, and the consumed energy of $MCM_{k}$ to receive data from $MCH_{i}$ is estimated as $S_{T}\times P_{Rx,i,k}/R_{i,k}$.

Furthermore, the power used by the $MCH_{i}$ battery to transfer data to $MCM_{k}/SCH_{i}$ is expressed as $P_{Tx,i,k}$. Thus, $S_{T}\times P_{Tx,i,k}/R_{i,k}$ [28,29] gives the energy consumed by $MCH_{i}$ to convey the content to $MCM_{k}/SCH_{i}$. As a result, the $P_{Tx}$ derivation for $MCH_{i}$ is given as

(8) $$P_{Tx}=P_{Tx,i,k}=P_{tx,ref,i,k}+P_{t,i,k}$$

where $P_{tx,ref,i,k}$ denotes the *i*th $MCH_{i}$ source circuitry power consumption during transmission via the communication link with the *k*th $MCM_{k}$, while the power transmitted over the air interface from $MCH_{i}$ to $MCM_{k}$ links is denoted by the variables $P_{t,i,k}$. A multi-hop link was used to establish a communication between the $MCH_{i}$ and the $MCM_{k}/SCH_{i}$. Thus, the total energy consumption $E_{C_{k}}$ can be estimated as [29]:

(9) $$E_{c_{k}}=ST\sum_{\begin{array}{c} i\neq k\\ i=1,2,\cdots \left|C_{k}\right|\\ k\in C_{k} \end{array}}\left(\frac{\Gamma_{k}P_{Tx,i,k}+P_{Rx,i,k}}{R_{i,k}}+\frac{P_{Rx,i}}{R_{i}}\right)$$

The *i*th $MCH_{i}$ correspond to the consumed energy to receive data from the RS on the significant link (wireless link) in the first-term links, whereas the second-term links correspond to $MCH_{i}$ energy consumed to transmit the data to $MCH_{k}$ in its cluster $C_{k}$ through D2D communication. The variable $\Gamma_{k}$ differentiates between unicasting and multicasting. Furthermore, each UE has different data to send over the unicasting uplink. With shorter-distance connectivity among UEs, the $MCM_{k}/SCH_{i}$ has residual energy to connect with the $MCH_{i}$, which may deliver received information to the RS in the uplink and enhance the efficiency of the energy transfer. For each cluster $C_{k}$, the equivalent data is delivered to $MCM_{k}/SCH_{i}$ in the downlink.

Therefore, multicasting or unicasting over long-distance and short-distance networks is used. Thus, in D2D communication from the $MCH_{i}$ to $MCM_{k}/SCH_{i}$ with short-range unicasting, $\Gamma_{k}$ = 1. For the multicast short-range effect, the summation effect $\left(\Gamma_{k}=1/\left|C_{k}\right|-1\right)$ in Equation (9) is compensated, since transmission takes place only once. Therefore, it is essential to mention that the harvested energy estimated in Equation (1) must be greater than or equal to the energy consumption in Equation (9) for $C_{k}$, and that leads to:

(10) $$\begin{array}{c} E\geq E_{C_{k}}\\ \tau_{1}T\eta \sum_{n=1}^{N}P_{n,RS.1}{\left|H_{n,RS-MCHi}\right|}^{2}\geq E_{C_{k}}\\ \sum_{n=1}^{N}P_{n,RS.1}{\left|H_{n,RS-MCHi}\right|}^{2}\geq \frac{E_{C_{k}}}{\tau_{1}T\eta } \end{array}$$

Assuming that each sub-carrier has the same power, i.e., $P_{1,RS.1}=P_{2,RS.1}=\cdots P_{N,RS.1}=P_{RS.1}$, we should have:

(11) $$P_{RS.1}\geq \frac{E_{C_{k}}}{\tau_{1}T\eta \sum_{n=1}^{N}P_{n,RS.1}{\left|H_{n,RS-MCHi}\right|}^{2}}$$

### 4.2. Outage Probability

Clustering techniques and D2D communication have recently received much attention due to their ability to improve wireless network connectivity while using less power during disaster circumstances. This section investigates the outage probability of the proposed wireless network. First, the first-hop link outage probability between the RS and MCHs is evaluated, followed by the second hop between $MCH_{i}$ and $MCM_{k}/SCH_{i}$. Where the distance between the RS and MCHs is expressed as $d_{RS-MCH_{i}}$ and the distance between the MCH and an intended $MCM_{k}/SCH_{i}$ is expressed as $d_{k}$, where i∈MCHs and $k\in MCM_{k}/SCH_{i}$. The D2D link outage probability between the MCH and MCMs could be estimated by [29]:

(12) $$P_{out}=1-exp\left\{-\zeta \left(\theta_{d},\alpha \right)\left(\rho_{rs}\lambda_{rs}d_{1}^{2}+\frac{\rho_{ch}\lambda_{ch}}{N}d_{2}^{2}\right)\right\}$$

where $d_{1}=d_{RS-MCH_{i}}$ and $d_{2}=d_{k}$, $\theta_{d}$ is the SIR threshold for D2D mode transmission, and the path-loss exponent is expressed as α, while $\zeta \left(\theta_{d},\alpha \right)$ can be expressed as:

(13) $$\zeta \left(\theta_{d},\alpha \right)=\frac{2\pi^{2}}{\alpha }CSC\left(\frac{2\pi }{\alpha }\right)\theta^{2}/\alpha$$

Concerning the D2D link transmission, the outage probability could occur if one at least of the connections of the RS to the MCH or the MCH to the MCM/SCH does not achieve the threshold value $\theta_{d}$.

**Figure 4:**
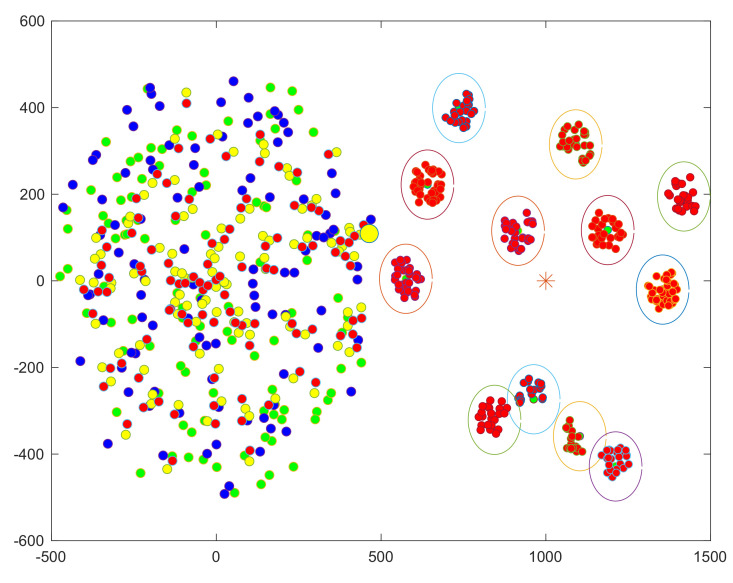
The visualization of clustering UEs in a disaster area where $C_{k}$ clusters were formed.

The location of the disaster area after forming clusters $C_{k}$ and selecting MCHs is illustrated in Figure 4. For each $C_{k}$, the D2D communication range has been plotted; green dots indicate the selected MCHs, red dots indicate MCMs/SCH in the right circular (dysfunctional area), whereas RS is represented by the large yellow dot in the left circle (functional area). However, the RS locates at $\left(x_{rs},y_{rs}\right)$, the MCH locates at $\left(x_{ch},y_{ch}\right)$, and the $MCM_{k}/SCH_{i}$ locates at $\left(x_{cm},y_{cm}\right)$; then, we can have

(14) $$\begin{array}{c} \underset{RS-MCH_{i}}{Distance}\\ \underset{MCH_{i}-MCM_{i,k}}{Distance} \end{array}=\left\{\begin{array}{c} d_{1}^{2}={\left(x_{ch}-x_{rs}\right)}^{2}+{\left(y_{ch}-y_{rs}\right)}^{2}\\ d_{2}^{2}={\left(x_{cm}-x_{ch}\right)}^{2}+{\left(y_{cm}-y_{ch}\right)}^{2} \end{array}\right.$$

Thus, the outage probability in Equation (12) could be rewritten as

(15) $$P_{out}=1-exp\left\{-\rho_{rs}\lambda_{rs}\zeta \left(\theta_{d},\alpha \right)f\left(x_{ch},y_{ch}\right)\right\}$$

where

(16) $$\begin{array}{cc} f\left(x_{ch},y_{ch}\right)= & {∥x_{rs}-x_{ch}∥}^{2}+{∥y_{rs}-y_{ch}∥}^{2}+\\ & \Lambda \left({∥x_{ch}-x_{cm}∥}^{2}+{∥y_{ch}-y_{cm}∥}^{2}\right) \end{array}$$

and Λ can be expressed as:

(17) $$\Lambda =\frac{\rho_{MCH_{i}}\lambda_{MCH_{i}}}{N\rho_{RS}\lambda_{RS}}$$

where $\lambda_{MCH_{i}}$ is the MCHs density, $\rho_{MCH_{i}}$ is the power transmitted by the MCHs, $\rho_{RS}$ is the RS load, and $\lambda_{RS}$ is the density of the RS. Since the selection of MCHs among UEs for every cluster is carried out on the basis of the CFM to balance the power consumption and improve the network capacity. Therefore, here we are determining the optimal location of the RS, which minimized $P_{out}$, and that can be achieved by.

(18) $$\begin{array}{cc} \left(x_{rs}^{o},y_{rs}^{o}\right)= & arg\underset{\left(x_{rs},y_{rs}\right)}{min}P_{out}\\ & =arg\underset{\left(x_{rs},y_{rs}\right)}{min}f\left(x_{r},y_{r}\right) \end{array}$$

By considering partial differentiation of $f\left(x_{rs},y_{rs}\right)$ with regard to $x_{rs}$ and $y_{rs}$ separately and equating it to zero, we can achieve the optimal RS location as:

(19) $$\left(x_{rs}^{o},y_{rs}^{o}\right)=\left(\frac{x_{bs}+\Lambda x_{ch}}{1+\Lambda },\frac{y_{bs}+\Lambda y_{ch}}{1+\Lambda }\right)$$

Since the optimal location of the RS is determined, the probability of an outage could be expressed as:

(20) $$\begin{array}{cc} P_{out} & \left(d,d_{o}\right)\\ & =1-exp\left\{-\rho_{rs}\lambda_{rs}\zeta \left(\theta_{d},\alpha \right)\left(\frac{\Lambda d^{2}}{1+\Lambda }+\left(1+\Lambda \right)d_{o}^{2}\right)\right\} \end{array}$$

where the distance between the optimal RS location and the $MCH_{i}$ donated as *d*, and the distance between other RS location and the optimal RS location $\left(x_{o},y_{o}\right)$ donated as $d_{o}$. Thus, we notice from Equations (18) and (19) that the RS is a circle centred at $\left(x_{o},y_{o}\right)$. Consequently, the path loss will affect the signal strength when the distance between the RS and MCH becomes larger. Therefore, the circular radius of the RS can be used to assess the success probability. If satisfactory, we can deduce that the $MCH_{i}$ can receive the RS coverage signal, and RS at the optimum location will be utilized for further communication to connect the wireless network in the disaster area.

### 4.3. The Communication Performance within Clusters

The received SNR at the MCMk/SCHi should be greater than the threshold value to ensure the correct decoding of the network receivers [30]. Consequently, the $MCM_{k}/SCH_{i}$ could communicate with the MCH using D2D communication.

According to the preceding definition, the expected received signals at the $MCM_{k}/SCH_{i}$ can be represented as follows when the *i*th MCH sends wireless signals:

(21) $$y_{i,k}=d_{i,k}^{-\alpha }\sqrt{G_{i,k}}P_{MCH}+\sigma_{0}$$

where $y_{i,k}$ represents the received wireless signal from the *i*th MCH, $P_{MCH}$ is the transmit power of the $MCH_{i}$, and $\sigma_{0}$ represents the additive noise at the MCHs that is assumed to be identically and independently distributed following complex Gaussian with zero-mean and $N_{0}$ variance, i.e., $\sigma_{0}∼\mathrm{CN}\left(0,N_{0}\right)$. Thus, the instant SINR received by the $MCM_{k}/SCH_{i}$ can be estimated as:

(22) $$y_{i,k}=\left(P_{MCH}G_{i,k}d_{i,k}^{-\alpha }\right)/\left(\sigma^{2}B_{t}\right)$$

$B_{t}$ is the total bandwidth, and $G_{i,k}$ represents the channel gain between the $MCH_{i}$ and the $MCM_{k}/SCH_{i}$. Thus, the link outage probability between the $MCH_{i}$ and the $MCM_{k}/SCH_{i}$ is represented as:

(23) $$\begin{array}{cc} P_{out}= & P\left(y_{min}>y_{k}\right)=P\left(\frac{y_{min}B_{t}\sigma_{0}}{P_{MCH}d_{i,k}^{-\alpha }}>G_{i,k}\right)\\ & =\int_{0}^{\left(-\left(y_{min}B_{t}\sigma_{0}\right)/P_{MCH}d_{i,k}^{-\alpha }\right)}exp\left(-x\right)dx\\ & =1-exp\left(\frac{y_{min}B_{t}\sigma_{0}}{P_{MCH}d_{i,k}^{-\alpha }}\right) \end{array}$$

The cluster’s D2D communication outage probability occurs when the link between the $MCH_{i}$ to $MCM_{k}/SCH_{i}$ in full-duplex mode is terminated. Therefore, the outage capacity is the maximum data rate achievable without an outage. As a result, the cluster’s D2D outage capacity is expressed as:

(24) $$\begin{array}{cc} C_{out,i,k}= & \left(1-P_{out,i,k}\right)B_{t}log_{2}\left(1+y_{min}\right)\\ & =e^{\left(\frac{y_{min}B_{t}\sigma_{0}}{P_{MCH}d_{i,k}^{-\alpha }}\right)}B_{t}log_{2}\left(1+y_{min}\right) \end{array}$$

where $C_{out,i,k}$ is based on the distance between the $MCH_{i}$ and the $MCM_{k}/SCH_{i}$ in each cluster $C_{k}$ and the bandwidth $B_{t}$ for D2D communication.

We assume that the $MCM_{k}/SCH_{i}$ receives the multicast signals from $MCH_{i}$ within the same time slot. Thus, the outage capacity of the multicast channel depends on the transmission rate for each $MCM_{k}/SCH_{i}$. Accordingly, $C_{out,i,k}=min\left\{C_{out,1},C_{out,2},\cdots \cdots C_{out,k}\right\}$.

## 5. Simulation Results and Discussion

This section presents the simulation results of the proposed framework, which considers the disaster scenario illustrated in Figure 1 to demonstrate its effectiveness. The simulation assumes that the operational BS transmits its signal at maximum power to extend signal coverage to the disaster area by using the RS as a relay at the edge of its coverage area. To connect the MCH in the disaster area, the RS transmits its signal power by employing $P_{Tx,RS}$ = 9 W and a bandwidth of $B_{t}$ = 10 MHz. Moreover, Table 1 details the simulation parameters that were used.

Notably, the received signal power at the destination UE is affected by path loss, which impacts the UE’s ability to harvest energy. As a result, it is critical to place the RS in the best possible location to receive good signal power at the MCHs. Consequently, we will discuss the optimal location of the RS, the wireless network efficiency in harvesting energy effectively, and the signal coverage outage probability.

**Figure 5:**
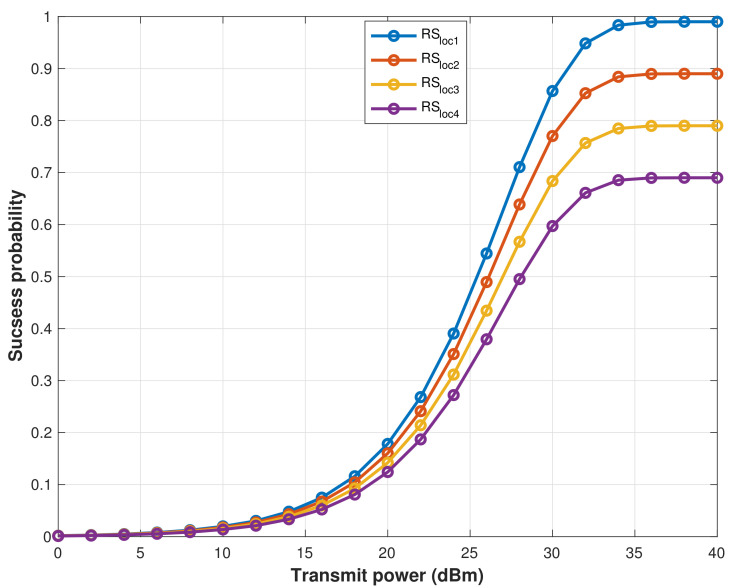
The success probability when RS transmits its signal by different power values at different locations.

Figure 5 shows the signal success probability when the RS transmits its signal using differing power values and is received at the furthest distance MCH from the different RS locations. The four candidate locations of the RS are $RS_{loc1}$, $RS_{loc2}$, $RS_{loc3}$, and $RS_{loc4}$, which are based on the greatest SINR achieved from the BS. As can be seen in Figure 5, $RS_{loc1}$ at the location of $\left(x_{rs},y_{rs}\right)$ achieved appropriate results in terms of received signal strength. Therefore, it is ideal for establishing a wireless communication link with MCHs, transmitting data, and transferring energy.

**Figure 6:**
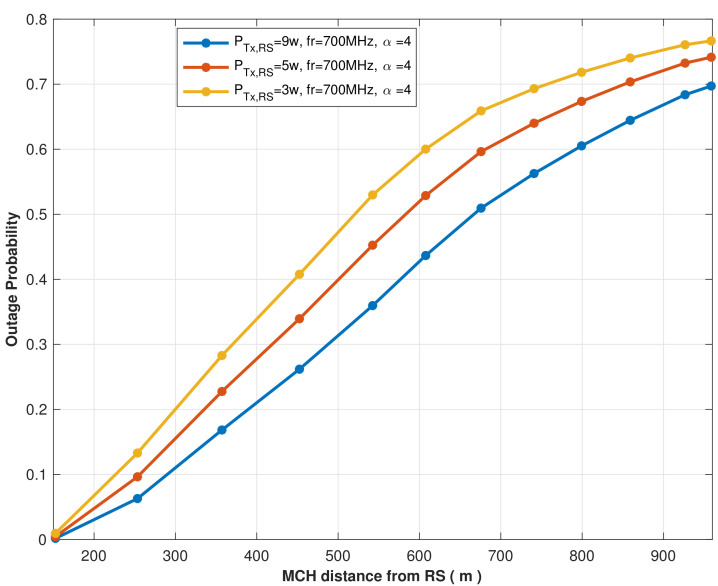
The outage probability vs. distance between MCHs and the optimal location of the RS when it transmits its signal by different power.

The outage probability vs. the MCHs distance from the RS is evaluated in Figure 6 to ensure appropriate power is used to transfer energy from the RS to the MCHs considering the optimal location of the RS $RS_{loc1}$. The investigation considers that the RS transmits its signal using 3 W, 5 W, and 9 W to transfer its energy to MCHs and utilizes 700 MHz as an operational frequency. Thus, the simulation results showed that the clustered UEs’ total outage probability increases as the distance to the RS increases. In the scenario where the RS transmits $P_{Tx,RS}$= 9 W, the outage probability gradually increases up to 70% at the most distant cluster, whereas it reached 78.5% when the RS transmits $P_{Tx,RS}$= 3 W, which is to be expected because the more transmitting power, the lower the probability of an outage. Generally, path loss occurs when the signal travels farther from the transmitter, so the outage occurs. The proposed RS-assisted wireless network strategy and the UAV-assisted wireless network approach are examined in the context of the disaster area illustrated in Figure 4, where 12 clusters have formed, and 12 UEs have been assigned as MCHs. The UAV altitude has been set at 100 m and it is transmitting its signal power using 5 W, which is assumed to be at the center of the disaster area. By assessing the outage probability of establishing links between MCHs using these two approaches, we can ensure the proposed strategy’s efficiency in terms of the energy harvesting outage probability at the 1st hop (RS to MCHs and UAV to MCHs).

**Figure 7:**
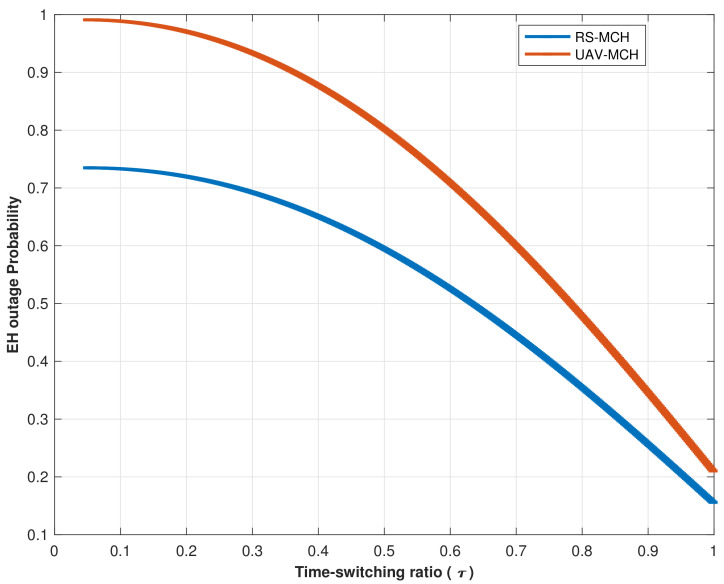
Comparison of EH outage probability versus time switching ratio (τ) for RS and UAV links.

Figure 7 assessed the EH outage probability vs. time switching factor (τ). The simulation findings reveal that as the number of time switching factors increases, the overall EH outage probability decreases. To put it another way, the higher the number of (τ), the lower the probability of EH failure during the transmission block time (T).

The RS model’s EH outage probability performance is significantly superior to the UAV model’s EH outage probability due to the enhanced channel quality associated with the RS model and the high transmitting power employed by the RS to establish links between the RS and MCHs. Furthermore, the UAV interferes with MCH signals, increasing the probability of an EH outage within its range.

**Figure 8:**
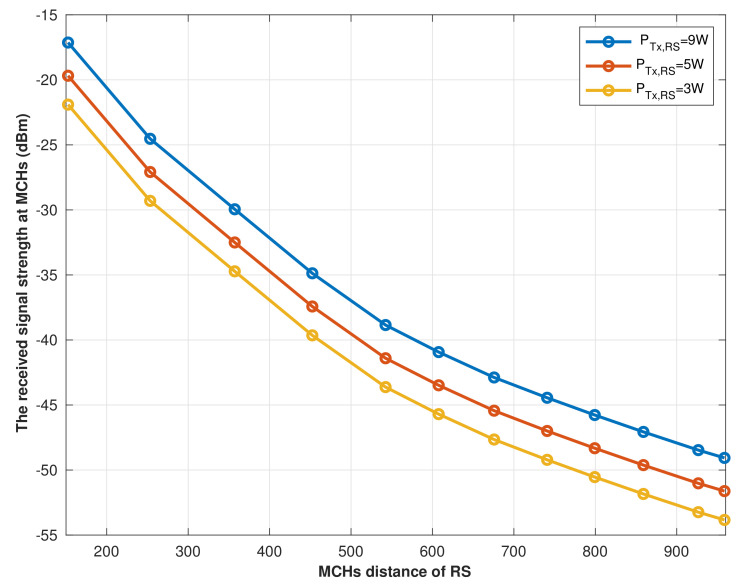
The received signal strength at $MCH_{i}$.

The transmission signal power that the RS uses to establish a successful connection with the MCH from its optimal location is investigated. As a result, Figure 8 illustrates the received signal at MCHs while the RS transmits at various power levels to ensure the quality of service of each cluster. Therefore, according to reference [31], the MCHs’ reference sensitivity is estimated in dBm based on the BS noise figure (NF), the bandwidth, and SNR required to reach the threshold throughput, which could be estimated as:

$$Receiver-sensitivity_{dBm}=-174+noisefigure+10log_{10}\left(Bandwidth_{\left(Hz\right)}\right)+SNR_{\left(dB\right)}$$

The result shown in Figure 8 indicates that as the distance between the MCHs and RS increases, the signal strength decreases, even though we are including the weakest signal that MCHs could be able to identify and process. Furthermore, when the RS transmits the signal with $P_{Tx,RS}$ = 9 W, the farthest MCH receives a signal power of $P_{Rx,MCH}$ = −68 dBm, whereas −74 dBm is received when the RS transmits with 3 W. As a result, it is essential to emphasize that higher received power at the MCH improves system capacity and gains more efficient energy harvesting.

**Figure 9:**
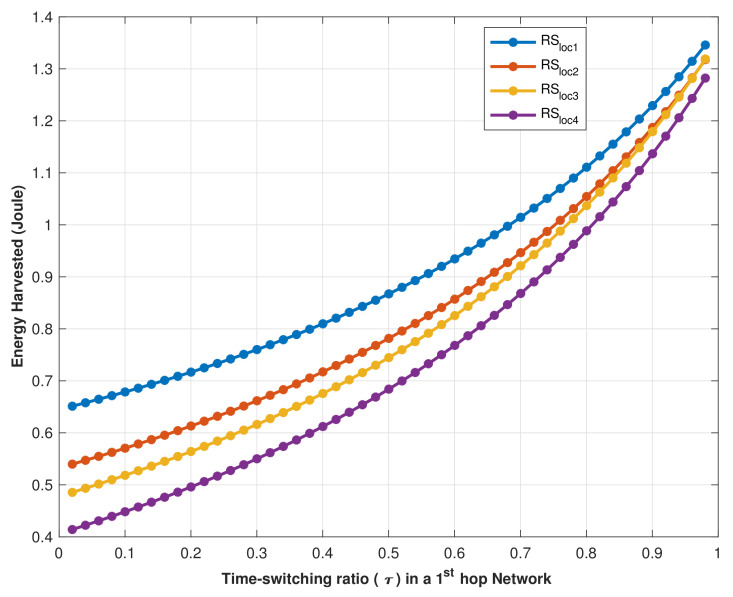
EH vs. time switching ratio (τ) at different locations of RS.

The path loss affects signals transmitted by the RS, even when MCHs are located in line of sight (LoS) with the RS, which has been observed from the strength of the received signal at the MCHs that are shown in Figure 8, which are affected vastly by the RS location and its signal transmitting power. Therefore, we investigated the impact of the RS’s location on the efficiency of energy harvesting by MCHs. Figure 9 showed the harvested energy vs. time switching ratio (τ) in the 1st hop (RS to MCHs) when the RS transmitted its signal power from four different locations ($RS_{loc1}$, $RS_{loc2}$, $RS_{loc3}$, and $RS_{loc4}$). According to the simulation result, EH increases as τ increases where 0<τ<1. However, in practice, τ cannot be set to 1 because it means that no communication data is transmitted. Thus, the RS at the location $RS_{loc1}$ is the optimal location where it gives better signal coverage, as shown in Figure 5, and efficient energy harvesting.

Since the received signal at the MCHs is affected by RS transmission signal power when the large-scale path loss considers the distances between the MCHs and RS while the bandwidth is fixed, so thus the energy harvested at the MCHs is also affected. Therefore, the RS transmission signal power is expected to affect EH performance since higher transmit power is required to compensate for increasing the distances between $RS-MCH_{i}$ and more hops between $MCH_{i}-MCM_{k}$.

**Figure 10:**
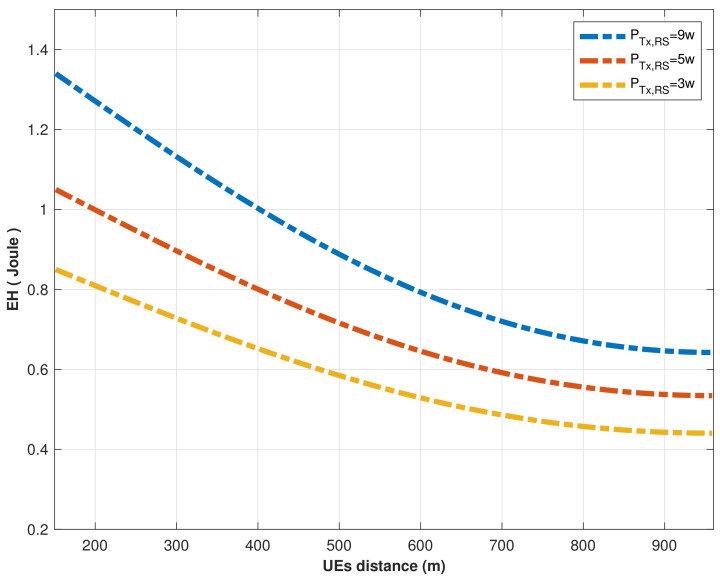
EH vs. MCHs distance from RS at different signal power transmitting by RS.

Accordingly, we investigated the probability of efficient energy harvesting for MCHs utilizing the RS’s optimal location, where Figure 10 shows an examination of energy harvesting at MCHs when the RS transmits its signal by different power values at its optimal location. The EH steadily decreases as a function of the MCHs as the distance increases. When $P_{Tx,RS}$ = 9 W, EH decreased from 1.32 to 0.62 joules as the distance increased from 152 to 945 m, while EH decreased from 0.84 to 0.49 joules as the distance increased from 152 to 945 m when $P_{Tx,RS}$ = 3 W, demonstrating that the RS transmission signal power had an impact on the EH since as the distance increased, a higher transmit power would be necessary.

**Figure 11:**
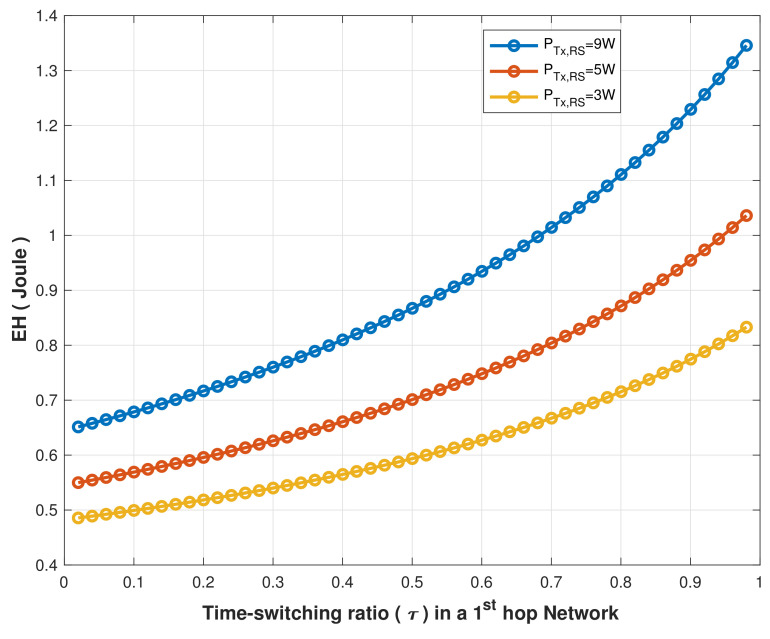
EH performance vs. time switching ratio (τ) at different $P_{Tx,RS}$ values.

Furthermore, we investigated the amount of energy harvested by MCHs at various time switching ratios (τ) when the RS is in its optimal location and transmits its signal at different power levels, as shown in Figure 11.

The EH steadily increases as the τ value increases. However, when $P_{Tx,RS}$ = 9 W, EH increases from 0.652 to 1.323 joules as the τ increases from 0.02 to 0.9. In comparison, EH increases from 0.49 to 0.84 joules as the τ increases from 0.02 to 0.9 when $P_{Tx,RS}$ = 3 W, demonstrating that the RS transmission signal power had an impact on the EH as the time switching ratio τ increased. Thus, a higher transmit power would be necessary to harvest a more significant amount of energy.

**Figure 12:**
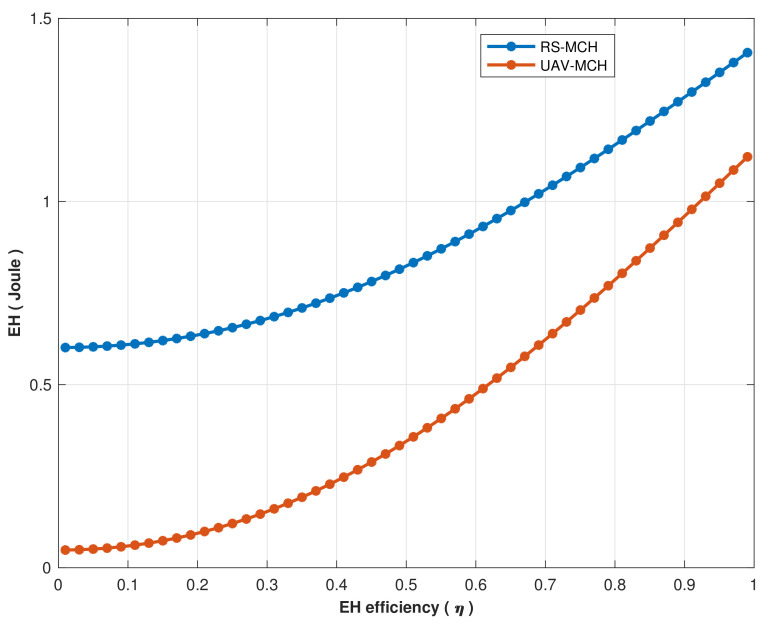
EH performance vs. energy harvesting efficiency (η) for RS and UAV.

The performance of EH versus energy harvesting efficiency (η) for RS and UAV communication is simulated in Figure 12. According to the simulation result, the RS scenario obtained 0.52 joule at η = 0 and gradually increased as the η value increased to achieve 1.49 joule when η = 1, whereas the UAV scenario achieved 0.12 joule and gradually increases as η value increases to achieve 1.18 joule when η = 1. Hence, The RS-assisted wireless link scenario maximizes EH approximately 20.8% better than the UAV link scenario through MCHs.

As a result, the EH performance in the RS scenario is superior to that in the UAV communication situation. The aforementioned is attributed to the slight loss of signal power received at the UEs and the considerable propagation path gain between the RS and MCHs. Further, EH in the UAV communication scenario is lower than the RS scenario due to the size of the disaster area compared to the UAV maximum cover range and the random-distributed UEs at spaces apart. The suggested framework ensures the continuity of wireless signal coverage in the disaster area.

Additionally, the framework utilizes wireless communication links assisted by an CFT and D2D communication, used to reduce UEs’ required transmission power to transfer their information, extend the network coverage area, and improve network spectral efficiency.

**Figure 13:**
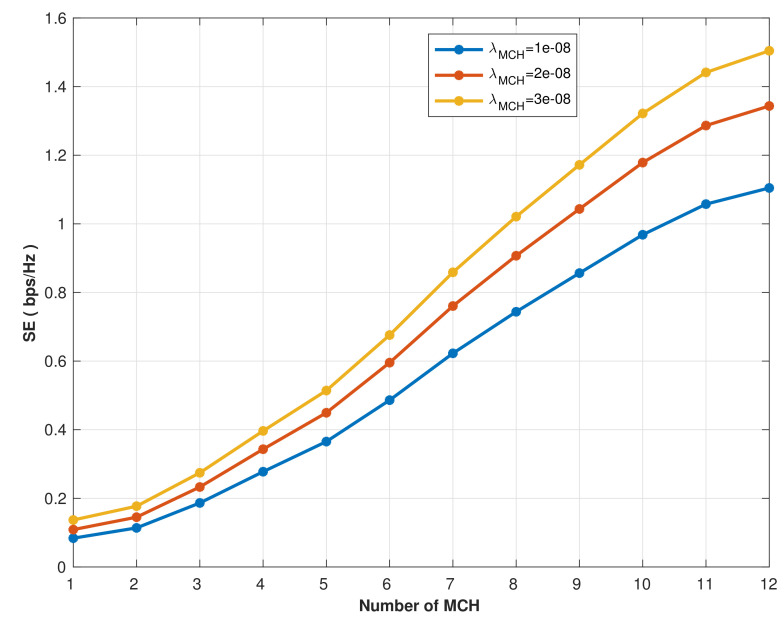
Spectral efficiency vs. MCH number with different density.

The network’s spectral efficiency performance with various MCH densities is shown in Figure 13. Due to the varying MCH densities and the efficient reuse of radio resources, as the MCHs’ numbers increase, the spectral efficiency increases, affecting network coverage. However, higher MCH densities improved spectral efficiency in the network scenario under investigation. The spectral efficiency increases from 0.14 bps/Hz to 1.1 bps/Hz when the MCHs are increased from 1 to 12 at MCH density $\lambda_{MCH}=1e^{-08}$.

Similarly, at MCH densities $\lambda_{MCH}=2e^{-08}$ and $\lambda_{MCH}=3e^{-08}$, the spectral efficiency improves from 0.15 bps/Hz to 1.38 bps/Hz and from 0.16 bps/Hz to 1.5 bps/Hz, respectively. Thus, a higher spatial density of MCHs, which is based on cluster formation, can serve more $MCM_{k}/SCH_{i}$ while maintaining the same spectral efficiency of the system. Furthermore, unlike the UAV model, the proposed communication system’s performance is unaffected by an increase in the number of clusters since the signal power used for the communication to cover the disaster area is stable and fixed.

**Figure 14:**
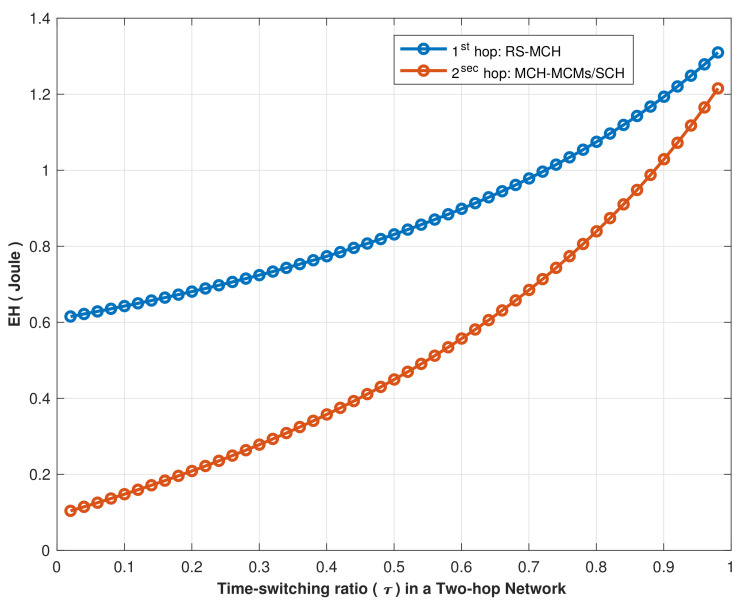
EH vs. time switching factor (τ) for a two-hop wireless network.

Figure 14 illustrated the energy harvesting performance for different time slots in two-hop EH strategies, $RS-MCH_{i}$ and $MCH_{i}-MCM_{k}/SCH_{i}$. According to the simulation results, it is noticeable that the EH in the second-hop connection (within the cluster) is less than in the first-hop connection (RS to MCHs) due to the transmission signal power used by the RS, which was as expected.

**Figure 15:**
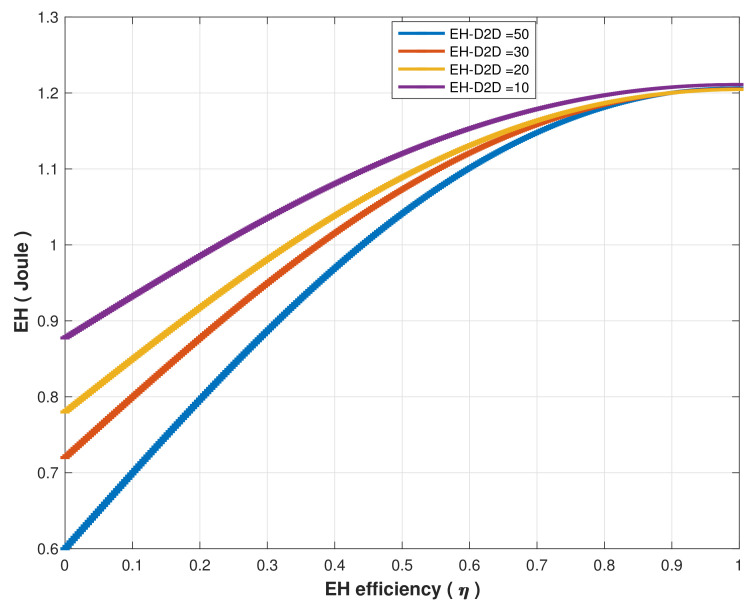
EH vs. energy harvesting efficiency (η) at different D2D distances.

Energy harvested versus the energy harvesting efficiency is estimated when the D2D distance between MCMs/SCH is 20, 30, 40, and 50 m. As demonstrated in Figure 15, generally, EH increases as the EH efficiency increases. Whereas the EH amount is less as the UE’s sparsity distance increases since the received SINR is affected by interference. In other words, due to decreased UE density, as the sparsity distance increases, D2D communication interference affected the received signal power, which affected the EH at the MCMs/SCH. The simulation result shows that when the distance between MCH and MCMs/SCH exceeds 30 m, EH performance is affected as the EH efficiency is set to less than 0.9.

## 6. Conclusions

In this article, WCN as a public safety network was enhanced and implemented. We provided a framework for a robust network in an emergency or disaster. The target was to offer UEs in disaster areas the best and most efficient communication, allowing them to communicate from the non-functional to the functional areas. As a result of the energy harvesting advantages of the proposed framework, MCMs, SCHs, and MCHs were able to function longer in critical scenarios, such as disasters. Furthermore, our proposed framework introduces a new stage to the provisioning phase for network survivability in a network failure. The proposed framework could be integrated with other restoration and protection approaches to improve network resilience in the aftermath of a disaster and provide better connectivity. Future research directions include an anticipation that the proposed framework can be enhanced to be suitable for application in a dynamic scenario (normal situation).

## Figures and Tables

**Table 1:** Parameters for simulation.

Parameter	Value
Radius of the cell (m)	500
*N* UEs’ average number	400
Number of clusters $C_{k}$	12
D2D main cluster (MC) range (m)	100
D2D sub-cluster (SC) range (m)	50
Maximum transmission power for the D2D link (mW)	100
Bandwidth $B_{t}$ (MHz)	10
Path-loss exponent α	4
Size of data content $S_{T}$	1–2 Mbit
Maximum transmit power $P_{TX,RS}$	40 dBm
Maximum transmit power $P_{TX,MCH}$	24 dBm
EH efficiency η	0–1
Time switching ratio τ	0.02–0.9
